# Integrated miRNA–Target Gene Analysis Reveals Complementary Molecular Biomarkers and Regulatory Networks in Pediatric Neuroblastoma

**DOI:** 10.3390/ijms27188139

**Published:** 2026-09-12

**Authors:** Karine Fiorentin, Solange S. Ferreira, Mayara O. Ruthes, Daiane Rosolen, Fernanda C. B. Berti, Hadelle H. Hassmann, Ana Paula Kuczynski, Lúcia de Noronha, Hellen G. G. Santos, Cleanderson R. Fidelis, Aline S. Fonseca, Luciane R. Cavalli

**Affiliations:** 1 Instituto de Pesquisa Pelé Pequeno Príncipe, Curitiba 80250-060, PR, Brazil; karine.fiorentin@aluno.fpp.edu.br (K.F.); solange.ferreira@professor.fpp.edu.br (S.S.F.); mayara.ruthes@aluno.fpp.edu.br (M.O.R.); daiane.rosolen@pelepequenoprincipe.org.br (D.R.); fernanda.berti@pelepequenoprincipe.org.br (F.C.B.B.); cleanderson@servidor.uepb.edu.br (C.R.F.); aline.fonseca@pelepequenoprincipe.org.br (A.S.F.); 2 Faculdades Pequeno Príncipe, Curitiba 80230-020, PR, Brazil; 3 Hospital Pequeno Príncipe, Curitiba 80250-060, PR, Brazil; hadelle.habitzreuter@hpp.org.br (H.H.H.); ana.kuczynski@hpp.org.br (A.P.K.); 4 Departamento de Biologia Celular e Molecular e Patologia Experimental, Pontifícia Universidade Católica do Paraná, Curitiba 80215-901, PR, Brazil; lucia.noronha@pucpr.br; 5 Laboratório de Bioinformática e Genômica Clínica, Instituto Carlos Chagas, Fiocruz, Curitiba 81350-010, PR, Brazil; hellen.santos@fiocruz.br; 6 Departamento de Estatística, Universidade Estadual da Paraíba, Campina Grande 58429-500, PB, Brazil; 7 Department of Oncology, Lombardi Comprehensive Cancer Center, Georgetown University, Washington, DC 20007, USA

**Keywords:** neuroblastoma, pediatric cancer, microrna, biomarkers, molecular characterization, precision oncology

## Abstract

Neuroblastoma is a heterogeneous pediatric malignancy with marked molecular diversity and variable clinical behavior. Although current risk stratification incorporates clinical and genomic factors, additional biomarkers are needed to improve tumor characterization and advance precision oncology. The present study investigated the expression of miR-141-3p, miR-150-5p, miR-181a-5p, and miR-182-5p and their predicted target genes in pediatric neuroblastoma. Tumor samples from 58 neuroblastoma and 13 ganglioneuroma patients were compared with 41 adjacent non-tumor tissues, while an independent cohort of 17 frozen tumors (13 neuroblastomas and 4 ganglioneuroblastomas) was used for integrated miRNA–target gene analyses. Diagnostic performance, functional enrichment, integrated analyses, and clinicopathological associations were evaluated. All four miRNAs were predominantly downregulated in tumors, with miR-150-5p and miR-181a-5p showing the most consistent reduction. A combined panel of miR-141-3p, miR-150-5p, and miR-181a-5p discriminated tumor from adjacent non-tumor tissues with 80.7% accuracy. miR-141-3p and miR-150-5p were associated with age at diagnosis, and miR-181a-5p was associated with overall survival. Functional enrichment implicated these miRNAs in neural crest development, stemness, neuronal differentiation, and major oncogenic pathways. Integrated analyses revealed reduced BCL2 expression in high-risk tumors and a positive correlation between miR-181a-5p and MYCN. These findings support these miRNAs as complementary molecular biomarkers and provide insights into miRNA-mediated regulatory networks in neuroblastoma.

## 1. Introduction

Neuroblastoma (NB) is the most common solid tumor of childhood, accounting for 8 to 10% of all childhood cancer cases and 12–15% of childhood cancer-related mortality [1,2]. Most cases are diagnosed during the first year of life, and less than 5% occur in children older than five years [1,3]. In Brazil, it is estimated that for 2026–2028, the annual incidence of pediatric cancer will be approximately 7560 new cases. The overall incidence of NB is 1 per 7000 live births, representing about 500 new cases of NB diagnosed annually in Brazil [4].

NB is a malignant neuroblastic tumor arising from primitive sympathetic ganglion cells and belongs to a spectrum of tumors that also includes ganglioneuroblastoma (GNB), which demonstrates intermediate malignant potential, and ganglioneuroma (GN), a benign tumor [5]. Clinically, NB is a highly heterogeneous disease, with tumors that range from those that regress spontaneously to those presenting as metastatic disease, which can be present at diagnosis in approximately 50% of patients [6,7,8,9]. Prognosis is determined by a combination of clinicopathological and biological features, including age at diagnosis, disease stage, histopathologic characteristics, and key molecular alterations such as *MYCN* amplification, tumor ploidy, and segmental chromosomal abnormalities (SCA), including 1p and 11q deletions and 17q gain [10,11,12]. Additional recurrent gene alterations include *ALK* mutations or amplification, *TERT* rearrangements, *ATRX* deletions, and *PHOX2B* mutations [13,14,15,16]. These molecular events play a central role in risk stratification and therapeutic decision-making and have been incorporated into international treatment guidelines [17,18] as well as in the development of targeted therapeutic strategies [19,20]. More recently, immunotherapeutic approaches have significantly improved outcomes in high-risk disease, particularly the incorporation of anti-GD2 monoclonal antibodies and CAR-T cells into multimodal treatment regimens, which has been associated with improved survival in children with high-risk NB [21,22].

In addition to genetic alterations, epigenetic dysregulation is an important component of NB pathogenesis and contributes to tumor heterogeneity, differentiation, and clinical behavior. Epigenetic mechanisms implicated in NB include aberrant DNA methylation, histone modifications, chromatin remodeling and dysregulation of non-coding RNAs (ncRNAs), which collectively influence gene expression programs involved in tumor development and progression [23,24,25]. Aberrant DNA methylation patterns and changes in chromatin regulatory mechanisms have been associated with the NB tumor phenotype, *MYCN* status, disease progression, and clinical outcome [24,26,27]. Among these epigenetic regulatory mechanisms, distinct classes of ncRNAs, including microRNAs (miRNAs), long non-coding RNAs (lncRNAs), and circular RNAs (circRNAs), play biologically significant roles in NB [28,29,30,31,32], consistent with their pivotal role in neural crest development and differentiation [33,34].

miRNAs, small endogenous RNAs that regulate gene expression [35], play a central role in cancer through the regulation of oncogenes and tumor suppressors, impacting key signaling pathways and biological cellular processes [36,37]. Under physiological conditions, miRNA expression is tightly regulated through coordinated transcription, processing, and post-transcriptional mechanisms, which can themselves be influenced by epigenetic processes such as DNA methylation and chromatin regulation [38].

In NB, dysregulated miRNA expression, often associated with *MYCN* amplification, epigenetic alterations, or SCA, has been linked to tumor development, progression, chemoresistance and poor clinical outcomes, supporting their potential roles as NB biomarkers and therapeutic targets [39,40,41]. Among these, miR-141-3p [42], miR-150-5p [43,44,45,46,47], miR-181a-5p [48,49], and miR-182-5p [50,51,52,53] were selected based on previous evidence demonstrating their involvement in cancer-related processes relevant to NB, including proliferation, apoptosis, migration, invasion, metastasis, and treatment response. In NB specifically, miR-141-3p has been associated with cell proliferation, migration, and cisplatin chemosensitivity [54], whereas miR-181a has been implicated in tumor cell growth, invasion, and metastasis, with ABI1 identified as one of its regulatory targets [55,56,57]. In contrast, the roles of miR-150-5p and miR-182-5p have been more extensively characterized in other malignancies, where they regulate proliferation, apoptosis, invasion, metastasis, and tumor-associated signaling pathways [44,50,51,52,53], while their biological and clinical relevance in NB remains less defined. These observations provided the rationale for investigating these four miRNAs and their associated target gene networks in pediatric NB.

Based on these observations, it was hypothesized that miR-141-3p, miR-150-5p, miR-181a-5p, and miR-182-5p and their corresponding target genes are differentially expressed in NB and that their expression patterns are associated with molecular pathways and clinicopathological features relevant to tumor behavior and clinical outcome. To test this hypothesis, the present study investigated the expression of these miRNAs and their putative target genes by integrating expression profiling with target gene, pathway, and clinicopathological analyses to characterize their biological relevance and potential as complementary molecular biomarkers for this disease.

## 2. Results

### 2.1. miRNA Expression Analysis Between Neuroblastoma (NB) and Ganglioneuroma (GN) and Between Tumor and Adjacent Non-Tumor (ANT) Samples

The expression of the four-miRNA panel was initially evaluated relative to the endogenous control gene (RNU48). A consistent pattern of predominantly low expression across all four miRNAs was observed in most of the formalin-fixed paraffin-embedded (FFPE) tumor samples (NB + GN = 71) (Figure S1). miR-141-3p and miR-182-5p, evaluated in 49/71 (69%) and 52 (73.4%) samples, respectively, were consistently downregulated. In contrast, miR-150-5p and miR-181-5p, evaluated in 58 (81.7%) and 56 (78.9%) samples, respectively, showed increased expression in a few cases.

**Figure 1:**
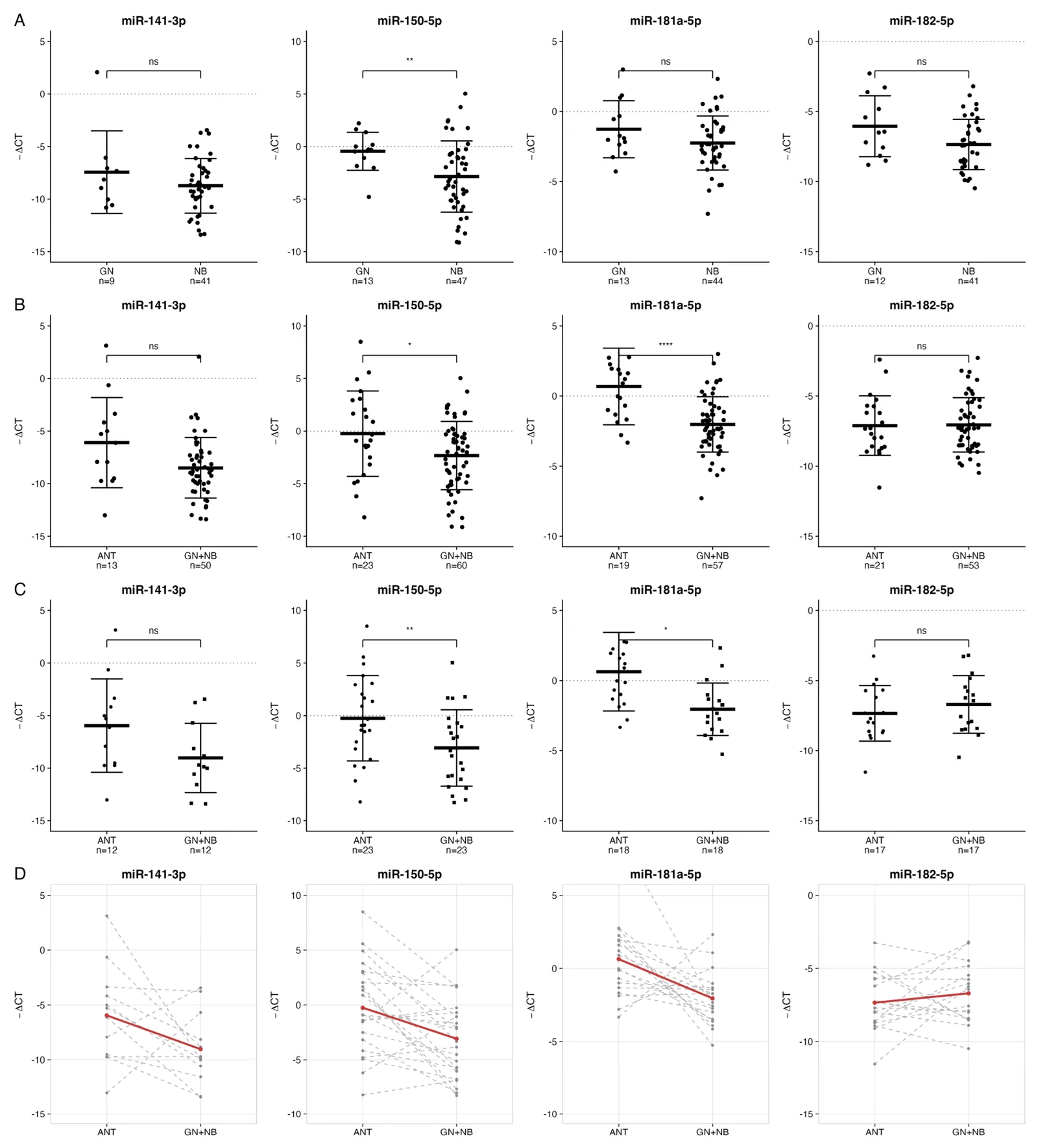
Relative expression of the miRNA panel in neuroblastic tumors and adjacent non-tumor (ANT) tissues. (**A**) Comparison of miR-141-3p, miR-150-5p, miR-181a-5p, and miR-182-5p expression between ganglioneuroma (GN) and neuroblastoma (NB) tumor samples. (**B**) Group-level comparison between ANT tissues and grouped GN and NB tumor samples. (**C**) Comparison between matched ANT and tumor tissues obtained from the same patients. (**D**) Paired expression profiles of matched ANT and tumor samples. Each point represents an individual sample. In panel (**D**), gray lines connect samples obtained from the same patient, and the red line represents the mean expression profile. T, tumor; n, number of samples or matched pairs, as indicated; ns, not significant. * *p* < 0.05, ** *p* < 0.01, **** *p* < 0.0001.

Comparison of miRNA expression between NB and GN tumor tissues revealed significantly lower miR-150-5p expression in NB compared to GN (*p* = 0.0088), whereas no significant differences were observed for the other miRNAs (Figure 1A). However, the comparison of the tumors grouped (NB + GN) with ANT tissue samples revealed significance for miR-150-5p and miR-181a-5p (*p* = 0.0445 and *p* = 0.00008) and a close-to-significant value for miR-141-3p (*p* = 0.0534) (Figure 1B).

Paired analysis of tumor tissues and corresponding ANT confirmed these findings, with miR-150-5p and miR-181a-5p significantly downregulated in tumor samples (GN + NB) compared to matched ANT tissues (*p* = 0.0059; log2FC = −2.0913, and *p* = 0.0102; log2FC = −1.25815, respectively) (Figure 1C). Figure 1D provides an alternative visualization of the same paired ANT/tumor samples, illustrating the individual expression profiles and the overall expression trends. Consistent with the results shown in Figure 1C, miR-150-5p and miR-181a-5p were significantly more upregulated in ANT tissues compared to their matched tumor samples, whereas miR-141-3p displayed a similar trend without reaching statistical significance.

The expression of the miRNAs was further evaluated by ROC analysis to assess their discriminatory ability, individually and in combination, to distinguish tumor samples from ANT samples (unpaired samples). Individual analyses showed AUC values ranging from 0.5112 (miR-182-5p) to 0.8033 (miR-181a-5p). Among the combined models, miR-150-5p + miR-181a-5p achieved the highest AUC (0.7063), although this value remained slightly lower than that observed for miR-181a-5p alone (AUC = 0.8033) (Table 1).

Finally, based on the AUC analyses and application of the Youden index, optimal cut-off points were determined for each individual miRNA and for the combined miRNA models. The cut-off values varied across miRNAs (miR-141-3p = 0.263158873, miR-150-5p = 0.360634, miR-181a-5p = 0.242793, and miR-182-5p = 0.287524). Using these thresholds, the single-classifier model for miR-141-3p achieved the highest classification accuracy (79.03%), whereas the model for miR-182-5p showed the lowest accuracy (71.2%). The multiple-classifier model comprising miR-141-3p + miR-150-5p + miR-181a-5p yielded a cut-off point of 0.120909663 and correctly classified 80.7% of the samples (Table S1).

### 2.2. miRNA Expression Analysis Between Pre- and Post-Chemotherapy Tumor Samples

**Figure 2:**
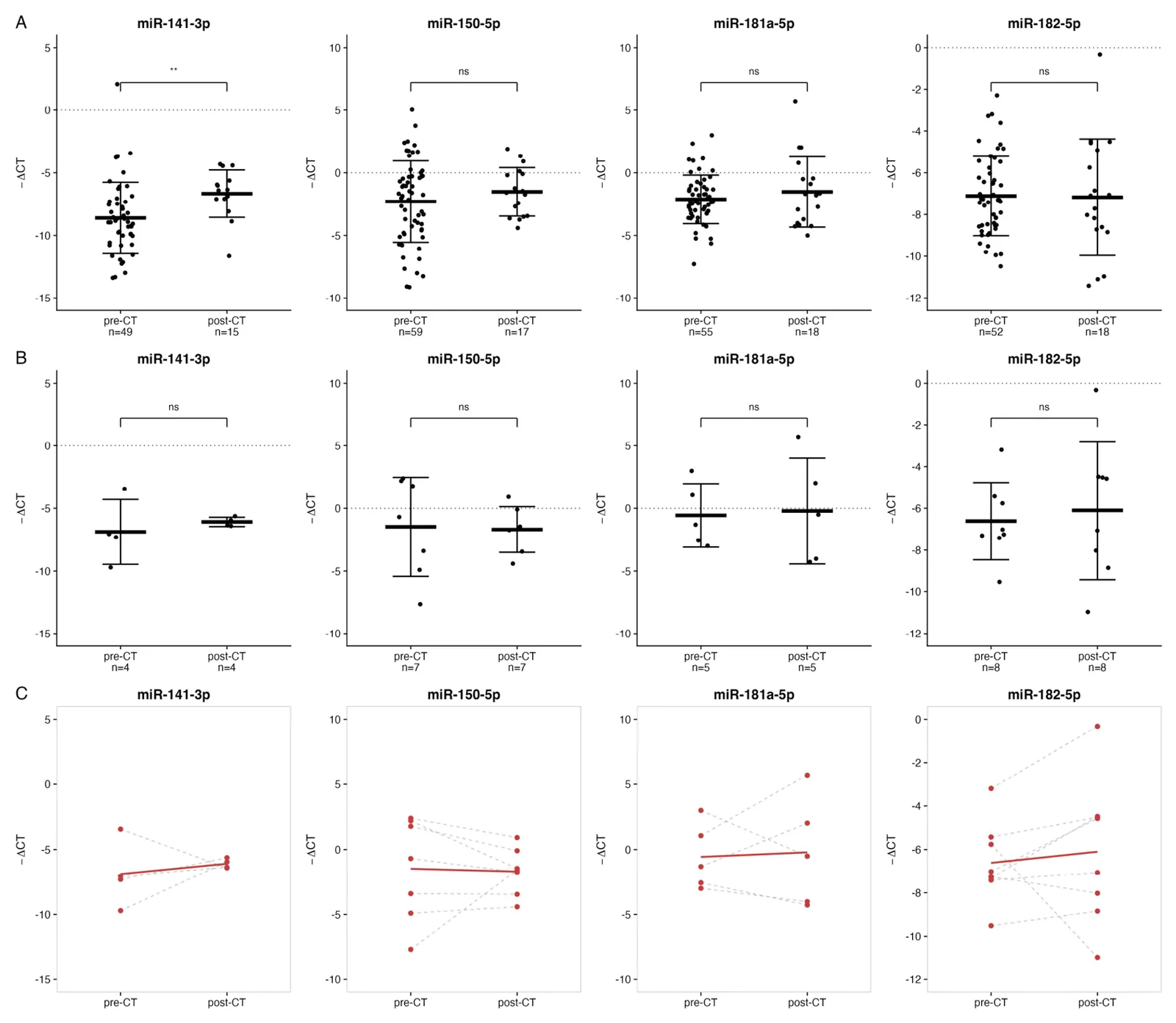
Relative expression of the miRNA panel in tumor samples pre- and post- chemotherapy (CT). (**A**) Group-level comparison of miR-141-3p, miR-150-5p, miR-181a-5p, and miR-182-5p expression between pre-CT and post-CT tumor samples. (**B**) Comparison between matched pre- and post-CT samples obtained from the same patients. (**C**) Paired expression profiles of matched pre- and post-CT samples. Each point represents an individual sample. In panel (**C**), gray lines connect samples obtained from the same patient, and the red line represents the mean expression profile. CT, chemotherapy; n, number of samples or matched pairs, as indicated; ns, not significant. ** *p* < 0.01.

Comparison of miRNA expression between pre- and post-CT tumor samples revealed, in unpaired analyses, that only miR-141-3p was significantly upregulated following CT (*p* = 0.002; FC = 3.74) (Figure 2A). In contrast, paired analyses showed no significant differences in miRNA expression between treated and untreated samples (Figure 2B), suggesting limited chemotherapy-related effects on basal miRNA expression. Relative expression analysis demonstrated a trend toward increased miR-141-3p expression post-CT, whereas no significant differences were observed for miR-150-5, miR-181a-5p, or miR-182-5p (Figure 2C). These findings, however, should be interpreted with caution due to the limited number of matched pre- and post-CT samples.

ROC curves were generated, and AUC values were calculated to assess the ability of the evaluated miRNAs to discriminate pre- and post-CT samples, individually and in combination (Table 2). Individual analyses showed AUC values ranging from 0.5128 (miR-182-5p) to 0.7660 (miR-141-3p), whereas combined models showed AUC values ranging from 0.5240 (miR-150-5p + miR-181a-5p + miR-182-5p) to 0.6280 (miR-141-3p + miR-182-5p). These results demonstrated the robust discriminatory potential of miR-141-3p for distinguishing samples according to treatment status.

The cut-off values determined using the AUC values and Youden index varied among the evaluated miRNAs (miR-141-3p = 0.264535, miR-150-5p = 0.19661, miR-181a-5p = 0.268898, and miR-182-5p = 0.267372). Among the single-classifier models, miR-182-5p achieved the highest classification performance (78.6%), followed closely by miR-141-3p (78.13%) in discriminating samples according to treatment phase (pre- and post-CT). Conversely, miR-150-5p showed the lowest classification performance (47.37%) (Table S2).

### 2.3. Association of miRNA Expression with Clinicopathological Features and Overall Survival

miRNA expression levels in tumor tissues were evaluated in relation to the main prognostic characteristics of patients with NB and GN, including age at diagnosis, sex, tumor stage, *MYCN* amplification status, Shimada classification, and risk group (Table S3). Significant associations were observed between age at diagnosis and the expression of miR-141-3p (*p* = 0.0258) and miR-150-5p (*p* = 0.0075). No significant associations were identified between miRNA expression and sex, tumor stage, *MYCN* amplification, Shimada classification, or risk group.

**Figure 3:**
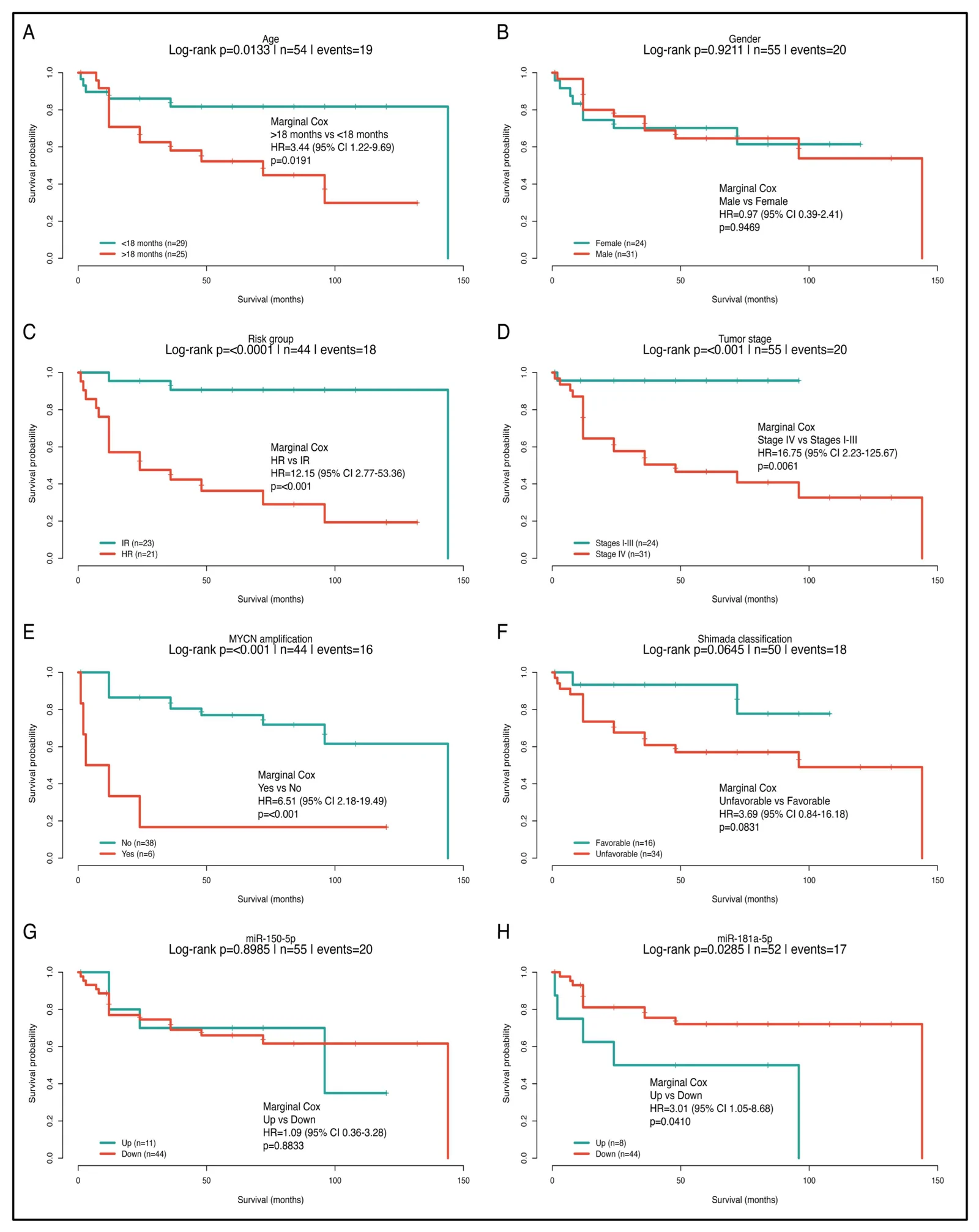
Kaplan–Meier survival curves stratified by age (**A**), gender (**B**), risk group (**C**), tumor stage (**D**), *MYCN* amplification (**E**), Shimada classification (**F**), miR-150-5p expression status (**G**) and miR-181a-5p expression status (**H**). Differences between survival curves were assessed using the log-rank test. Hazard ratios (HRs) were estimated using marginal Cox proportional hazards models. Vertical marks indicate censored observations. HR, high risk; IR, intermediate risk.

Kaplan–Meier analyses confirmed the expected associations between established prognostic factors and overall survival (OS). Patients diagnosed at >18 months of age, those with high-risk disease, stage IV tumors, or *MYCN* amplification had significantly poorer overall survival than their respective comparison groups (Figure 3A–E). Although patients with an unfavorable Shimada classification also showed reduced survival, this association did not reach statistical significance (Figure 3F). The Kaplan–Meier analysis of the expression of the miRNAs in relation to OS was performed for miR-150-5p and miR-181a-5p, considering that miR-141-3p and miR-182-5p only presented with expression downregulation. No evidence of a difference in OS was observed between the miR-150-5p expression groups (log-rank *p* = 0.8985; upregulated vs downregulated: HR = 0.92; 95% CI: 0.31–2.78; *p* = 0.8833) (Figure 3G). In contrast, OS differed significantly according to miR-181a-5p expression status (log-rank *p* = 0.0285). Patients with downregulated miR-181a-5p expression had a 67% lower estimated hazard of death than those with upregulated expression (HR = 0.33; 95% CI: 0.12–0.96; *p* = 0.0410) (Figure 3H).

### 2.4. Target Gene Selection and Enrichment Functional Analysis

Analysis of predicted target genes revealed multiple cellular signaling pathways potentially regulated by the studied miRNAs. Using curated interactions from the TarBase and KEGG databases, 132 significantly (*p* < 0.01; FDR < 0.05) enriched signaling pathways were observed (pathway union) (Figure S2). These pathways formed a highly interconnected network controlling central biological processes in NB, such as cell stemness, differentiation, survival, and invasion. Notably, miR-181a-5p was involved in the highest number of pathways (105/132 = 79.5%), followed by miR-182-5p (73 = 55.3%) and miR-150-5p (52 = 39.4%), whereas miR-141-3p was associated with a smaller number of pathways (22 = 16.7% of pathways). The 15 top pathways involving at least three of the miRNAs studied are summarized in Table 3.

A second heatmap comprising 55 biological processes (*p* < 0.05) involving at least one of the studied miRNAs was generated using the Gene Ontology (GO) category (union function) (Figure S3). Among these, 15 biological processes were regulated by at least two miRNAs (Diana tools/miRPath, v.4.0) (Table 4). Consistent with the pathway analysis described above, miR-181a-5p (41/55 = 74.5%) was involved in the highest number of biological processes, followed by miR-182-5p (23 = 41.8%). miR-141-3p (18 = 32.7%) and miR-150-5p (7 = 12.7%) were associated with a smaller number of biological processes.

Target gene selection for each miRNA was performed using the TarBase v.9 and miRTarBase v.9 databases. Only target genes present in both databases were included. For each miRNA, a high number of predicted target genes was initially identified: miR-181a-5p (n = 7282), miR-182-5p (n = 8055), miR-141-3p (n = 2113), and miR-150-5p (n = 1342). Therefore, only target genes supported by strong experimental evidence (luciferase assay, qPCR, or Western blot) were selected for further analysis. This second filtering step resulted in 28 target genes (Table S4), with the highest numbers observed for miR-182-5p (n = 13), miR-181a-5p (n = 11), and miR-141-3p (n = 10). miR-150-5p showed the lowest number of experimentally validated target genes supported by strong evidence (n = 4). Of the total 28 target genes, seven were shared by the miRNAs: *BCL2* and *PTEN* (miR-141-3p, miR-181a-5p, miR-182-5p), *VEGFA* (miR-141-3p, miR-150-5p, miR-181a-5p), *CCND1* (miR-141-3p, miR-182-5p), *CDKN1A* and *TIAM1* (miR-181a-5p, miR-182-5p), and *TP53* (miR-150-5p, miR-181a-5p).

A protein–protein interaction (PPI) network was constructed with proteins of the selected seven target genes, in addition to the MYCN protein, with a minimum confidence score of 0.7 (high-confidence interactions), showing 29 nodes with a highly significant PPI enrichment (*p* < 1.0 × 10^−16^) (Figure S4). Three major clusters were identified: cluster 1, consisting of four proteins (EDN1, PF4, PTPN11, and VEGFA); cluster 2, consisting of six proteins (CCND1, CCNE2, CDK4, E2F3, PPARA and RB1); and cluster 3, consisting of 19 proteins (ATM, AURKA, BARD1, BCL2, BRCA1, CDK2, CDKN1A, CHEK2, FBXW7, HRAS, KRAS, MAPK1, MCL1, MYB, MYCN, NRAS, PTEN, TIAM1 and TP53).

**Figure 4:**
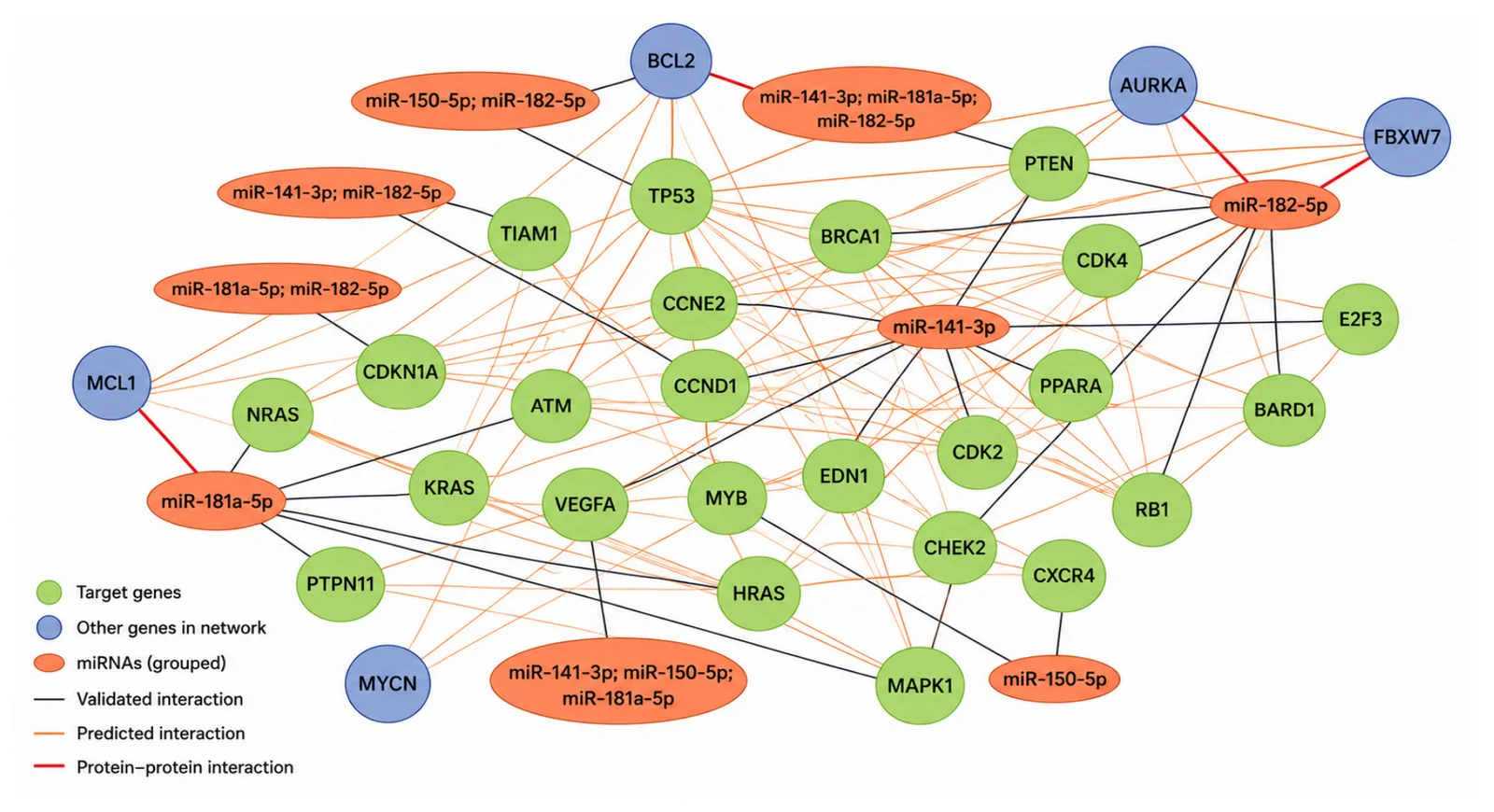
miRNA–mRNA network based on the target genes of miR-141-3p, miR-150-5p, miR-181-5p and miR-182-5p. mRNAs highlighted in green and proteins in blue were selected for experimental analysis in the tumor samples. Blue and red lines represent miRNA–mRNA and miRNA–mRNA (genes selected) interactions, respectively (Cytoscape v.3.10.4).

Subsequently, a miRNA–mRNA interaction network was constructed (Figure 4), demonstrating extensive interconnectedness among the identified miRNAs and target genes, with multiple shared regulatory interactions. Among the 28 target genes identified, four genes (*AURKA*, *BCL2*, *FBXW7*, and *MCL1*), in addition to *MYCN*, were selected for expression analysis in the 17 frozen neuroblastic tumor tissue samples (see below). This selection was based on the involvement of these genes in NB [58,59,60,61], their biological interactions within the network, and their potential functional relevance to tumor progression and survival pathways.

### 2.5. Select miRNAs and Target Genes Analyses

Overall, among the 17 samples of frozen tumor tissue analyzed (13 NB, 3 GNB, 1 GN), lower expression levels were observed for miR-141-3p, miR-181a-5p, and miR-182-5p. miR-150-5p showed increased expression in only two samples, whereas the remaining samples showed reduced expression relative to the endogenous control (Figure S5A). For the target genes, RT-qPCR expression analysis was successfully performed in 82.4% of the samples for *AURKA* and *BCL2*, 100% for FBXW7, 88.2% for *MCL1*, and 94.1% for *MYCN*. Overall, these genes showed reduced expression relative to the mean expression of the endogenous controls (*ACTB* and *GAPDH*) (Figure S5B).

Pearson correlation analysis was performed to determine the correlation between the expression of the miRNAs, target genes, and *MYCN*. A statistically significant moderate to strong positive linear correlation between miR-181a-5p and *MYCN* expression (r = 0.6175; r2 = 0.3813; *p* = 0.0108) was observed, suggesting coordinated expression between these molecules (Table S5).

### 2.6. Association of miRNA and Target Gene Expression with Clinicopathological Features and Overall Survival

In the NB, GNB, and GN frozen tumor samples, associations between miRNA and target gene expression and the main clinical–prognostic characteristics and follow-up data were investigated, including age at diagnosis, sex, tumor stage for NB cases, *MYCN* amplification status, and risk classification. Survival analyses for this set of samples were not performed due to the limited sample size and the lack of distinct expression groups, which precluded reliable estimation of associations with overall survival.

The number of samples included in each analysis varied according to the availability of expression and clinical data. For the miRNA panel, up to 17 ΔCt values were available for each marker, whereas the number of available values for the target genes varied among the genes: *AURKA* (n = 14), *BCL2* (n = 15), *FBXW7* (n = 17), *MCL1* (n = 15), and *MYCN* (n = 17). As shown in Table S6, no statistically significant associations were observed between the expression levels of the four miRNAs and the evaluated clinical or prognostic parameters. Similarly, most associations involving the target genes were not statistically significant. However, MYCN expression was significantly associated with *MYCN* amplification status (*p* = 0.0190; FC = 10.42), whereas BCL2 expression was significantly associated with risk group (*p* = 0.0072; FC = −2.75). None of the other variables showed statistically significant associations.

## 3. Discussion

The present study investigated the expression of miR-141-3p, miR-150-5p, miR-181a-5p, and miR-182-5p, selected based on their established involvement in cancer [42,43,44,45,46,47,48,49,50,51,52,53,62], including NB [54,55,56,57,63]. Overall, a predominantly reduced expression pattern was observed across the neuroblastic tumor samples for all four miRNAs. Among them, miR-150-5p and miR-181a-5p demonstrated the most consistent and biologically relevant findings, showing significant downregulation in tumor tissues relative to non-tumor samples.

Although functional analyses were not performed in the present study, the significantly reduced expression of miR-150-5p and miR-181a-5p in tumor samples supports their potential function as tumor suppressors. miR-181a-5p showed the highest discriminatory performance between tumor and non-tumor tissues, and logistic regression analysis of ROC/AUC demonstrated that the combined panel of miR-141-3p, miR-150-5p, and miR-181a-5p achieved the highest predictive performance, correctly classifying 80.7% of samples. However, this model was developed using a training dataset only, highlighting the need for validation in a larger and independent cohort.

The tumor suppressor role of miR-150-5p and miR-181a-5p is consistent with previous studies describing the involvement of these miRNAs in tumor suppression and regulation of cell proliferation and apoptosis in different cancer types. Studies in colorectal cancer [64] and hepatocellular carcinoma [65] demonstrated significantly lower miR-150-5p expression in tumor tissues compared to non-tumor tissues. Also consistent with our findings, a recent study using miRNA sequencing of plasma small extracellular vesicles (sEVs) in NB has identified miR-150-5p among a subset of differentially expressed miRNAs distinguishing patients with NB from healthy controls [63]. Mechanistic studies indicate that miR-150-5p exerts inhibitory effects on cell proliferation and invasion by targeting key oncogenic drivers [45,46,47,65]. Notably, miR-150-5p has been described as a regulator of *MYCN*-associated pathways, and its reduced expression may contribute to enhanced *MYCN* signaling [66,67], supporting the aggressive phenotype characteristic of high-risk NB. In relation to miR-181a-5p, studies in retinoblastoma, another pediatric tumor of neuroectodermal origin, have demonstrated reduced miR-181a-5p expression in tumor tissues and cell lines compared with normal retinal tissues. Functional restoration of this miRNA suppressed cell proliferation, migration, and invasion while enhancing apoptosis by directly targeting *NRAS*, supporting a tumor-suppressive role for the miR-181a-5p/*NRAS* axis in retinoblastoma [68]. In NB, however, most studies report that miR-181a-5p is highly expressed and associated with increased cell invasion and metastasis [55,69]. These contrasting findings suggest that the biological effects of miR-181a-5p may be tumor- and cellular-context-dependent, potentially reflecting differences in its target gene networks and downstream signaling pathways. Interestingly, another target gene regulated by miR-181-5p is *CCND1*, one of the 28 target genes identified in the PPI network (see below). While several miRNAs directly inhibit *CCND1* to restrain G1/S transition and proliferation in solid tumors, miR-181a has been shown to sustain CCND1 expression in multiple myeloma, where the miRNA is overexpressed, and its inhibition rather than its overexpression reduces CCND1 levels and induces cell cycle arrest [70]. Given that *CCND1*, located at 11q13, encodes a core regulator of the CDK4/6-RB axis and is a well-established oncogenic driver of proliferation across cancers, this tissue- and context-dependent relationship illustrates that the direction and mechanism of miR-181a-5p/CCND1 regulation cannot be assumed to be uniform across malignancies and reinforces the need for direct functional validation of this interaction in NB-derived models.

miR-182-5p did not show significantly different expression between tumor and non-tumor tissues; however, it demonstrated the highest classification performance according to chemotherapy phase. miR-182-5p belongs to the highly conserved miR-183/96/182 cluster (miR-183C), which has been implicated in the regulation of immunity, autoimmunity, and cancer-related processes [71]. Dysregulation of miR-183C members has been reported in several malignancies, particularly gastrointestinal cancers, where their expression has been investigated for its potential diagnostic and prognostic biomarker value [72]. The expression and activity of miR-183C members are influenced by several cancer-related signaling pathways, including BCL-2, p53 and PI3K/AKT signaling [73,74]. These pathways are also associated with miR-182-5p in the present analyses. These findings support the context-dependent biological and potential biomarker relevance of miR-182-5p and may help explain its discriminatory performance according to chemotherapy phase despite the absence of significant differential expression between tumor and non-tumor tissues.

Clinicopathological analyses further demonstrated that miR-141-3p and miR-150-5p were significantly associated with older age at diagnosis (>18 months), a well-established adverse prognostic factor in NB. In addition, miR-181a-5p expression was associated with overall survival, suggesting that these miRNAs may reflect distinct biological and clinical aspects of disease progression. Although these associations require further validation, they support the potential clinical relevance of the studied miRNAs as complementary molecular biomarkers in NB.

miRNAs are key regulators of chemoresistance and potential predictors of treatment response in NB [31,40,75]. Although limited clinical data in the present study did not allow for direct correlation with treatment outcomes, miR-141-3p showed significantly lower expression in pre-chemotherapy samples and strong discriminatory power. This result was consistent with the accuracy of this miRNA in correctly classifying 78% of the samples according to the chemotherapy phase. Its increased expression after treatment suggests a role in therapeutic response, potentially enhancing chemosensitivity. This effect may involve modulation of anti-apoptotic genes, such as *BCL2* and *MCL1*, and regulation of *FUS*, a DNA repair gene linked to apoptosis and treatment sensitivity, as previously demonstrated [54,76,77,78,79].

Functional enrichment and pathway analyses further supported the biological relevance of the selected miRNAs in NB pathogenesis, as they converge on key processes involved in neural crest development, stemness, neuronal differentiation, proliferation, survival, migration, and therapy response [80,81,82]. Axon guidance, neurotrophin signaling, and stem cell signaling pathways are particularly relevant given the developmental origin of NB [34,83], while PI3K-AKT, MAPK, ERBB, WNT, RAP1, and FOXO signaling constitute interconnected oncogenic networks that regulate cell proliferation, apoptosis, differentiation, stem cell maintenance, and therapeutic resistance [84,85,86,87]. In parallel, focal adhesion and proteoglycans in cancer pathways suggest that the identified miRNA targets may also influence tumor–microenvironment interactions, cytoskeletal remodeling, invasion, and metastatic behavior [88,89].

Following target gene identification, a PPI network was constructed with 28 relevant proteins from the identified target genes, including BCL2, CCND1, CDKN1A, PTEN, TIAM1, TP53 and VEGFA. This network, based on high-confidence interactions, exhibited significantly more PPIs than expected for a random set of proteins of similar size and genomic distribution, indicating that they are biologically connected and functionally associated.

Based on their previously reported relevance in NB [59,61,90,91,92,93], four target genes (*AURKA*, *BCL2*, *FBXW7*, and *MCL1*) were selected from the identified set, along with *MYCN*, for expression analysis in the 17 frozen neuroblastic tumor samples. These analyses revealed reduced expression levels of *AURKA*, *BCL2*, *FBXW7*, *MCL1*, and *MYCN* across tumor samples, with significant differences observed for BCL2 expression between low- and high-risk patient groups. Furthermore, MYCN expression was significantly higher in *MYCN*-amplified tumors, consistent with its well-established role as a major oncogenic driver in NB.

Although predicted regulatory relationships between miRNAs and their target genes were identified through bioinformatic analyses, co-expression analyses did not reveal significant correlations for most miRNA–gene pairs. This lack of direct correlation may reflect the complexity of miRNA-mediated regulation, which often involves indirect interactions, cooperative regulation by multiple miRNAs, and context-dependent gene expression patterns. Additionally, temporal differences between miRNA expression and downstream gene regulation may contribute to the absence of detectable correlations in bulk tumor analyses. Nevertheless, a significant positive correlation between miR-181a-5p and MYCN expression was identified, suggesting its involvement in MYCN-driven oncogenic networks and reinforcing its potential relevance in NB progression.

## 4. Materials and Methods

### 4.1. Sample Population

Formalin-fixed paraffin-embedded (FFPE) tumor tissues from pediatric patients diagnosed with neuroblastoma (NB; n = 58) and ganglioneuroma (GN; n = 13) were obtained from Hospital Pequeno Príncipe (HPP), Curitiba, Paraná, Brazil. The samples were collected between 2005 and 2014, with informed consent after approval by the HPP Ethics Committee (CAAE: 21683219.4.0000.0097). Of the 71 patients with FFPE tumor samples analyzed, 60 (NB = 47, GN = 13) were obtained pre- (pre-CT) and 11 (NB = 11) post-chemotherapy (post-CT). For eight cases, it was possible to obtain matched pre- and post-CT samples. Adjacent non-tumor (ANT) tissue samples for a subset of the patients (n = 41; NB = 38, GN = 3) were used as controls to determine the cancer specificity of miRNA expression.

A second set of tumor samples (NB = 13, GNB = 4) was collected at HPP between 2016 and 2021 with informed consent after approval from the HPP Ethics Committee. These consisted of frozen tissue samples, including 12 samples obtained pre-CT and five (stage IV patients) post-CT and hematopoietic stem cell transplantation.

All cases were classified according to the International Neuroblastoma Staging System (INSS) [94,95] and the Shimada Histopathological Classification System [96,97]. Risk group assessments were based on the International Risk Group (INRG) system [98,99]. The *MYCN* amplification status was determined using fluorescence in situ hybridization (FISH) [100]. Clinical and pathological information was obtained from pathology reports and medical records by the attending clinicians. Patients were followed from diagnosis until death or last clinical follow-up, with a follow-up of 2 to 7 years. All data were anonymized prior to being made available to the research team, in accordance with ethical regulations. These data are summarized in Table 5.

### 4.2. RNA Isolation and RT-qPCR Analysis

Tumor and non-tumor (ANT) tissue samples of FFPE were evaluated for the content of tumor cells by the pathologist prior to the molecular analysis. The selected areas, tumor and ANT, were punched from the blocks and deparaffinized with xylene and absolute ethanol. RNA was isolated using the RNA Recover All Total Nucleic Acid Isolation kit (Thermo Fisher Sci., Waltham, MA, USA). Frozen tissue samples were processed in liquid nitrogen, and the RNA was extracted using Trizol (Invitrogen/Thermo Fisher Sci., Waltham, MA, USA), according to the manufacturer’s recommendations.

RNA was reverse transcribed using the TaqMan MicroRNA Reverse Transcription kit and amplified by RT-quantitative PCR (RT-qPCR) using TaqMan probes for miR-141-3p (assay #000463), miR-150-5p (assay #000473), miR-181a-5p assay #000480), miR-182-5p (assay #002334), and RNU48 (assay #1006) (normalizer) (Applied Biosystems/Thermo Fisher Sci, Waltham, MA, USA), as previously described [41].

In the frozen tumor tissues, RT-qPCR analysis was also performed to evaluate the expression of the selected miRNA target genes and the MYCN gene. Specific TaqMan assays were used for each gene: AURKA (assay #984332), BCL2 (assay #2010408), FBXW7 (assay #1959375), MCL1 (assay #2002511), MYCN (assay #1996272), ACTB (assay #2032743) (normalizer), and GAPDH (assay #1688730) (normalizer) (Applied Biosystems/Thermo Fisher Sci).

RT-qPCR reactions were performed in triplicate using an ABI Prism^®^ 7500 Fast Sequence Detection System (Applied Biosystems/Thermo Fisher Scientific), as previously described [44]. Data are presented as mean ± SE. miRNA expression differences between groups were calculated using the 2-ΔΔCT method [101]. Statistical analyses were performed in GraphPad Prism v.8 [102] using the Mann–Whitney and Kruskal–Wallis tests. Co-expression between miRNAs and their target genes was assessed by Pearson’s correlation and linear regression analyses. *p* < 0.05 was considered statistically significant. All analyses were conducted using pre-CT samples only, except for those specifically comparing pre-CT and post-CT groups.

### 4.3. Target Gene Selection

miRPath v.3, available on the Diana tools platform (http://diana.imis.athena-innovation.gr/DianaTools/index.php, accessed on 19 February 2026) and based on the Kyoto Encyclopedia of Genes and Genomes (KEGG) (last accessed date: 19 February 2026), was used to perform the enrichment analyses of the biological functions and signaling pathways associated with the selected miRNAs. TarBase v.9 [103] (last accessed date: 19 February 2026) was used to generate heatmaps illustrating miRNA–mRNA interactions and to conduct gene union/pathway enrichment analyses. Pathways with *p* < 0.05 were considered statistically significant. miRTarBase v.9 [104] (last accessed date: 19 February 2026) was also used to identify experimentally validated target genes. Only target genes present in both TarBase v.9 and miRTarBase v.9 and miRNA–mRNA interactions supported by strong experimental evidence (luciferase assay, qPCR, or Western blot) were selected.

STRING v.12 [105] (last accessed date: 27 February 2026) was used to generate a protein–protein interaction (PPI) network with proteins encoded by the selected genes and the MYCN protein, which plays a key role in the prognosis of NB patients. A minimum required interaction score of 0.7 (high confidence) was applied, and disconnected nodes were removed from the network. The resulting network was subsequently imported into Cytoscape v.3.10.4 [106], where the four miRNAs analyzed were incorporated to generate a comprehensive miRNA–mRNA interaction network.

### 4.4. Receiver Operating Characteristic (ROC) Curve Analysis

ROC curve analysis was performed to determine the discriminatory power of the expression of the miRNAs between tumor tissue and ANT and between pre-CT and post-CT samples. GraphPad Prism 8.0 [102] was used to create the ROC curves and to calculate the area under the curve (AUC). Test sensitivity was plotted against 1 − specificity for binary classifiers. AUC values and confidence intervals of 95% were calculated for each miRNA and/or combinations of miRNAs.

In addition, R v.4.5.2 [107] and RStudio v.2026.1.1.403 [108] software were used to further evaluate the discriminatory performance of the miRNAs using a random-effects logistic regression model, allowing incorporation of repeated measures from the same individual (paired data). Both single-marker models (one miRNA) and a multiple-marker model (including all miRNAs) were fitted. Model performance was assessed based on the AUC of the corresponding ROC curves. The Youden index was applied to determine optimal probability cut-off values for sample classification (tumor vs. ANT), yielding estimates of sensitivity, specificity, and the frequency of true- and false-positive classifications.

### 4.5. Clinicopathological, Co-Expression, and Survival Analyses

The miRNA expression levels obtained from tumor tissues, excluding paired samples from the same patient, were evaluated according to clinical variables, including age at diagnosis, sex, tumor histopathology according to the Shimada classification, disease stage, MYCN amplification status, and clinical risk group classification. For categorical variables, statistical analyses were performed in GraphPad Prism v.8 [102] using the Mann–Whitney and Kruskal–Wallis tests to compare miRNA expression levels.

Pearson’s correlation coefficient was calculated in GraphPad Prism v.8 [102] to estimate linear correlations between miRNA expression and continuous variables. Co-expression between each miRNA and the selected genes was assessed separately using Pearson’s correlation coefficient and simple linear regression. The number of observations included in each analysis varied according to the combined availability of valid expression and clinical data. The miRNA expression levels obtained from frozen tissue samples were analyzed separately, considering that tissue material may affect miRNA expression [109,110].

For the survival analyses, overall survival (OS) was defined as the time from diagnosis to death from any cause. Patients who remained alive were censored at the date of their last available clinical follow-up. Kaplan–Meier curves [111] were used to estimate the survival functions, and differences between groups were assessed using the log-rank test [112]. Marginal Cox proportional hazards regression models [113] were fitted to evaluate the association of clinicopathological variables and miRNA expression in the FFPE cohort. Survival analyses according to miRNA or target gene expression were not performed in the frozen-tissue cohort because the limited sample size and the absence of clearly defined expression groups precluded reliable estimation. For all statistical analyses, a significance level of 5% was adopted.

## 5. Conclusions

Overall, the present study demonstrates that integrated analyses of miRNA expression, putative target genes, and functional enrichment provide new insights into the molecular landscape of pediatric NB and highlight the potential of miR-141-3p, miR-150-5p, miR-181a-5p and miR-182-5p as complementary molecular biomarkers. The significantly reduced expression of miR-150-5p and miR-181a-5p in tumor tissues supports their potential tumor-suppressive roles, although functional validation is required. Among the individual miRNAs, miR-181a-5p showed the highest discriminatory performance between tumor and non-tumor tissues, while the combined signature of miR-141-3p, miR-150-5p, and miR-181a-5p achieved the highest predictive performance, correctly classifying 80.7% of samples. In addition, the association of miRNA expression with chemotherapy phase and *MYCN* expression suggests their potential relevance for treatment monitoring and molecular risk stratification. Collectively, these findings support the further evaluation of miRNA-based signatures as complementary tools for the molecular characterization of NB and as potential adjuncts to histopathological and molecular assessment. Validation in larger independent cohorts, longitudinal studies and functional analyses will be required to establish their clinical utility.

## Figures and Tables

**Table 1:** ROC curve demonstrating the discriminatory performance of individual/combined miRNAs between tumor and ANT samples.

miRNAs	AUC	Error	IC95%	*p*-Value
miR-141-3p	0.6754	0.09072	0.4976 to 0.8532	0.0528
miR-150-5p	0.6435	0.07074	0.5048 to 0.7821	0.0440 *
miR-181a-5p	0.8033	0.05886	0.6880 to 0.9187	<0.0001 *
miR-182-5p	0.5112	0.07596	0.3624 to 0.6601	0.8809
miR-141-3p + miR-150-5p	0.6568	0.05320	0.5525 to 0.7611	0.0048 *
miR-141-3p + miR-181-5p	0.6925	0.05830	0.5782 to 0.8067	0.0010 *
miR-141-3p + miR-182-5p	0.5939	0.05672	0.4828 to 0.7051	0.1011
miR-150-5p + miR-181a-5p	0.7063	0.04933	0.6097 to 0.8030	<0.0001 *
miR-150-5p + miR-182-5p	0.5479	0.05389	0.4422 to 0.6535	0.3523
miR-181a-5p + miR-182-5p	0.5609	0.05977	0.4438 to 0.6780	0.2547
miR-141-3p + miR-150-5p + miR-181a-5p	0.6800	0.04397	0.5938 to 0.7662	<0.0001 *
miR-141-3p + miR-150-5p + miR-182-5p	0.5956	0.04430	0.5088 to 0.6825	0.0317 *
miR-141-3p + miR-181a-5p + miR-182-5p	0.5974	0.04741	0.5045 to 0.6903	0.0337 *
miR-150-5p + miR-181a-5p + miR-182-5p	0.5939	0.04617	0.5034 to 0.6844	0.0277 *
miR-141-3p + miR-150-5p + miR-181a-5p + miR-182-5p	0.6120	0.03963	0.5344 to 0.6897	0.0036 *

Legend: * AUC with significant *p*-value (*p* < 0.05).

**Table 2:** ROC curve demonstrating the discriminatory performance of individual/combined miRNAs between pre- and post-CT samples.

miRNAs	AUC	Error	IC95%	*p*-Value
miR-141-3p	0.7660	0.06769	0.6333 to 0.8987	0.0019 *
miR-150-5p	0.5720	0.06870	0.4374 to 0.7067	0.3689
miR-181a-5p	0.5139	0.08839	0.3406 to 0.6871	0.8600
miR-182-5p	0.5128	0.08660	0.3431 to 0.6826	0.8719
miR-141-3p + miR-150-5p	0.5987	0.05007	0.5006 to 0.6969	0.0908
miR-141-3p + miR-181-5p	0.5625	0.05336	0.4579 to 0.6671	0.2798
miR-141-3p + miR-182-5p	0.6280	0.05590	0.5184 to 0.7375	0.0276 *
miR-150-5p + miR-181a-5p	0.5456	0.05448	0.4388 to 0.6524	0.4151
miR-150-5p + miR-182-5p	0.5260	0.05729	0.4137 to 0.6383	0.6440
miR-141-3p + miR-150-5p + miR-181a-5p	0.5560	0.04340	0.4709 to 0.6410	0.2317
miR-141-3p + miR-150-5p + miR-182-5p	0.5767	0.04403	0.4904 to 0.6630	0.1020
miR-141-3p + miR-181a-5p + miR-182-5p	0.5559	0.04436	0.4689 to 0.6428	0.2308
miR-150-5p + miR-181a-5p + miR-182-5p	0.5240	0.04623	0.4334 to 0.6146	0.5993
miR-141-3p + miR-150-5p + miR-181a-5p + miR-182-5p	0.5498	0.03835	0.4746 to 0.6250	0.2159

Legend: * AUC with significant *p*-value (*p* < 0.05).

**Table 3:** Top 15 significantly enriched signaling pathways and corresponding target genes regulated by at least three of the miRNAs studied.

	KEGG Pathway	Merged *p*-Value	#Target Genes (per miRNA)	#miRNAs	miRNAs
1	Axon guidance	1.4973 × 10^−20^	36,35, 21, 14	4	miRs-181a-5p, 182-5p, 141-3p, 150-5p
2	Proteoglycans in cancer	2.23783 × 10^−17^	33, 37, 21,18	4	miRs-181a-5p, 182-5p, 141-3p, 150-5p
3	Pathways in cancer	4.29792 × 10^−15^	66, 59, 42, 31	4	miRs-181a-5p, 182-5p, 141-3p, 150-5p
4	Wnt signaling pathway	1.21758 × 10^−12^	24, 21, 17, 18	4	miRs-181a-5p, 182-5p, 141-3p, 150-5p
5	PI3K-Akt signaling pathway	2.06771 × 10^−12^	51, 40, 29, 21	4	miRs-181a-5p, 182-5p, 141-3p, 150-5p
6	Focal adhesion	3.48275 × 10^−12^	37, 30, 13	3	miRs-141-3p, 150-5p, 181a-5p, 182 5p
7	Growth hormone synthesis, secretion and action	1.46364 × 10^−11^	25, 22, 10	3	miRs-141-3p, 150-5p, 181a-5p, 182 5p
8	EGFR tyrosine kinase inhibitor resistance	7.26615 × 10^−11^	20, 14, 8	3	miRs-181a-5p, 182-5p, 150-5p
9	Neurotrophin signaling pathway	8.06204 × 10^−11^	26, 19, 9	3	miRs-181a-5p, 182-5p, 150-5p
10	MAPK signaling pathway	1.78186 × 10^−10^	44, 42, 28	3	miRs-181a-5p, 182-5p, 141-3p
11	ErbB signaling pathway	3.90605 × 10^−10^	18, 13, 10	3	miRs-181a-5p, 182-5p, 150-5p
12	Rap1 signaling pathway	3.79103 × 10^−10^	30, 33, 20	3	miRs-181a-5p, 182-5p, 141-3p
13	FoxO signaling pathway	2.66188 × 10^−9^	28, 17, 9	3	miRs-181a-5p, 182-5p, 150-5p
14	Prolactin signaling pathway	4.5862 × 10^−9^	19, 11, 6	3	miRs-181a-5p, 182-5p, 150-5p
15	Signaling pathways regulating pluripotency of stem cells	1.58336 × 10^−8^	25, 19, 12	3	miRs-181a-5p, 182-5p, 150-5p

**Table 4:** Top 15 significant biological processes and corresponding number of target genes regulated by at least two of the miRNAs studied.

	GO Category	Merged *p*-Value	#Target Genes (per miRNA)	#miRNAs	miRNAs
1	Protein binding	2.65 × 10^−42^	1166,1100,781,324	4	miRs-181a-5p,182-5p,141-3p,150-5p
2	DNA-binding transcription factor activity, RNA polymerase II-specific	9.99 × 10^−35^	134,111,94,37	4	miRs-181a-5p,182-5p,141-3p,150-5p
3	DNA-binding transcription factor activity	2.43 × 10^−31^	130,104,93,39	4	miRs-181a-5p,182-5p,141-3p,150-5p
4	DNA-binding transcription activator activity, RNA polymerase II-specific	2.01 × 10^−27^	78,72,56	3	miRs-181a-5p,182-5p,141-3p
5	RNA polymerase II cis-regulatory region sequence-specific DNA binding	4.19 × 10^−23^	99,81,72	3	miRs-181a-5p,182-5p,141-3p
6	Transferase activity	1.7 × 10^−22^	221,200,145	3	miRs-181a-5p,182-5p,141-3p
7	DNA binding	2.03 × 10^−22^	277,182	2	miRs-181a-5p,141-3p
8	Sequence-specific double-stranded DNA binding	2.53 × 10^−20^	89,68	2	miRs-181a-5p,141-3p
9	Protein kinase activity	2.14 × 10^−18^	88,66,48	3	miRs-181a-5p,182-5p,141-3p
10	Sequence-specific DNA binding	2.57 × 10^−18^	97,79,71	3	miRs-181a-5p,182-5p,141-3p
11	Protein serine/threonine kinase activity	4.88 × 10^−18^	77,53,39	3	miRs-181a-5p,182-5p,141-3p
12	Transcription regulatory region sequence-specific DNA binding	6.32 × 10^−16^	53,43,41	3	miRs-181a-5p,182-5p,141-3p
13	Chromatin binding	8.97 × 10^−16^	61,64,56	3	miRs-181a-5p,182-5p,141-3p
14	Kinase activity	2.51 × 10^−15^	104,84	2	miRs-181a-5p,182-5p
15	Transcription factor binding	8.71 × 10^−14^	60,48,22	3	miRs-181a-5p,182-5p,150-5p

**Table 5:** Clinicopathological and follow-up data of pediatric patients with neuroblastic tumors analyzed.

Clinical Variable	NB (n = 71 *)	GNB (n = 4)	GN (n = 13)
**A** **ge (n = 88)**			
<18 months	36 (50.7%)	1 (25%)	0
≥18 months	35 (49.3%)	3 (75%)	13 (100%)
**Sex (n = 88)**			
F	30 (42.3%)	2 (50%)	6 (46.2%)
M	41 (57.7%)	2 (50%)	7 (53.8%)
**Stages (n = 75)**			
1	16 (22.5%)	2 (50%)	NA
2	3 (4.2%)	1 (25%)	NA
3	8 (11.3%)	0	NA
4	38 (53.5%)	1 (25%)	NA
4S	6 (8.5%)	0	NA
**Stroma (n = 65)**			
Poor	43 (82.7%)	NI	0
Rich	9 (17.3%)	NI	13 (100%)
**Schwann cells (n = 65)**			
Absent	48 (92.3%)	NI	0
Present	4 (7.7%)	NI	13 (100%)
**Shimada (n = 65)**			
Favorable	17 (32.7%)	NI	13 (100%)
Unfavorable	35 (67.3%)	NI	0
**Risk groups (n = 62)**			
*LR*	30 (51.7%)	3 (75%)	NA
*IR*	10 (17.2%)	0	NA
*HR*	18 (31.03%)	1 (25%)	NA
***MYCN*** **amplification (n = 68)**			
Not Amplified	44 (80%)	2 (100%)	10 (90.1%)
Amplified	11 (20%)	0	1 (9.9%)
**Bone marrow infiltration** **(n = 56)**			
Absent	29 (53.7%)	0	1 (100%)
Present	25 (46.3%)	1 (100%)	0
**Recurrence (n = 72)**			
Negative	28 (47.5%)	4 (100%)	8 (88.9%)
Positive	31 (52.5%)	0	1 (11.1%)
**DFS (n = 85)**			
≥18 months	50 (73.5%)	4 (100%)	13 (100%)
<18 months	18 (26.5%)	0	0
**Survival status (n = 88)**			
Alive	45 (63.4%)	4 (100%)	13 (100%)
Death	26 (36.6%)	0	0

Legend: * The number of patients includes only one sample from each patient with paired pre- and post-CT samples. NB: neuroblastoma; GNB: ganglioneuroblastoma; GN: ganglioneuroma; NA: not available; LR: low risk; IR: intermediate risk; HR: high risk; CT: chemotherapy; DFS: disease-free survival.

## Data Availability

The raw data supporting the conclusions of this article will be made available by the authors on request.

## Supplementary Materials

The following supporting information can be downloaded at https://www.mdpi.com/article/10.3390/ijms27188139/s1.

## Abbreviations

The following abbreviations are used in this manuscript:

Gene list	Acronyms and full names
*ABI1*	Abl interactor 1
*ACTB*	Actin beta
*AKT*	AKT serine/threonine kinase
*ALK*	ALK receptor tyrosine kinase
*ATM*	ATM serine/threonine kinase
*ATRX*	ATRX chromatin remodeler
*AURKA*	Aurora kinase A
*BARD1*	BRCA1-associated RING domain 1
*BCL2*	BCL2 apoptosis regulator
*BRCA1*	BRCA1 DNA repair associated
*CCND1*	Cyclin D1
*CCNE2*	Cyclin E2
*CDK2*	Cyclin-dependent kinase 2
*CDK4*	Cyclin-dependent kinase 4
*CDK6*	Cyclin-dependent kinase 6
*CDKN1A*	Cyclin-dependent kinase inhibitor 1A
*CHEK2*	Checkpoint kinase 2
*E2F3*	E2F transcription factor 3
*EDN1*	Endothelin 1
*EGFR*	Epidermal growth factor receptor
*ERBB*	Erb-b receptor tyrosine kinase
*FBXW7*	F-box and WD repeat domain containing 7
*FOXO*	Forkhead Box O
*FUS*	FUS RNA-binding protein
*GAPDH*	Glyceraldehyde-3-phosphate dehydrogenase
*HRAS*	HRas proto-oncogene, GTPase
*KRAS*	KRas proto-oncogene, GTPase
*MAPK1*	Mitogen-activated protein kinase 1
*MCL1*	MCL1 apoptosis regulator, BCL2 family member
*MYB*	MYB proto-oncogene, transcription factor
*MYCN*	MYCN proto-oncogene, bHLH transcription factor
*NRAS*	NRAS proto-oncogene, GTPase
*PF4*	Platelet factor 4
*PHOX2B*	Paired like homeobox 2B
*PI3K*	Phosphatidylinositol-4,5-bisphosphate 3-kinase
*PPARA*	Peroxisome proliferator-activated receptor alpha
*PTEN*	Phosphatase and tensin homolog
*PTPN11*	Protein tyrosine phosphatase non-receptor type 11
*RAP1*	RAS oncogene family
*RB1*	RB transcriptional corepressor 1
*TERT*	Telomerase reverse transcriptase
*TIAM1*	TIAM Rac1-associated GEF 1
*TP53*	Tumor protein p53
*VEGFA*	Vascular endothelial growth factor A
*WNT*	Wnt family member
