# Supplementary material for: Integrated miRNA–Target Gene Analysis Reveals Complementary Molecular Biomarkers and Regulatory Networks in Pediatric Neuroblastoma
Source: Int J Mol Sci. 2026 Sep 12;27(18):8139. doi: 10.3390/ijms27188139 (PMC13607374; doi:10.3390/ijms27188139)
Supplement: Supplementary file 1 [file ijms-27-08139-s001.zip › ijms-4508985-supplementary.pdf]

Supplementary material

Supplementary tables

Table S1. Number of correctly labelled tumor (T) and adjacent non-tumor (ANT) samples [reference and prediction 0-0 (T) or 1-1 (ANT)], considering the cut-off value for each miRNA and the multiple miRNA model.

miRNA				
			Reference	
miR-141-3p			0 (T)	1 (ANT)
(Cut-off=0.263158873)	Prediction	0 (T)	41	6
		1 (ANT)	8	7
			Reference	
miR-150-5p			0 (T)	1 (ANT)
(Cut-off=0.360634)	Prediction	0 (T)	49	13
		1 (ANT)	10	10
			Reference	
miR-181a-5p			0 (T)	1 (ANT)
(Cut-off=0.242793)	Prediction	0 (T)	43	5
		1 (ANT)	12	14
			Reference	
miR-182-5p			0 (T)	1 (ANT)
(Cut-off=0.287524)	Prediction	0 (T)	11	7
		1 (ANT)	41	14
			Reference	
Multiple miR-141-3p+ miR-150-5p+ miR-181a-5p (Cut-off=0.120909663)			0 (T)	1 (ANT)
	Prediction	0 (T)	35	0
		1 (ANT)	11	11

Table S2. Number of correctly labelled pre- and post-QT samples [reference and prediction 0-0 (pre-CT) or 1-1 (post-CT)], considering the cut-off value for each miRNA and the multiple models.

miRNA				
			Reference	
miR-141-3p			0 (pre)	1 (post)
(Cut-off=0.263158873)	Prediction	0 (pre)	38	3
		1 (post)	11	12
			Reference	
miR-150-5p			0 (pre)	1 (post)
(Cut-off=0.360634)	Prediction	0 (pre)	20	1
		1 (post)	39	16
			Reference	
miR-181a-5p			0 (pre)	1 (post)
(Cut-off=0.242793)	Prediction	0 (pre)	43	11
		1 (post)	12	7
			Reference	
miR-182-5p			0 (pre)	1 (post)

(Cut-off=0.287524)	Prediction	0 (pre)	52	15
		1 (post)	0	3

Table S3. Association of miRNA expression with the prognostic parameters of the patients.

**Clinical variable**	**miR-141-3p**	**miR-150-5p**	**miR-181a-5p**	**miR-182-5p**
Age<br>(>18 months/<18 months)	**p=0.0258**<br>(n=60)	**p=0.0075**<br>(n=71)	p>0.9999<br>(n=68)	p=0.4608<br>(n=63)
Gender<br>(F/M)	p=0.2075<br>(n=60)	p=0.4006<br>(n=71)	p=0.8773<br>(n=68)	p=0.1149<br>(n=63)
Risk group<br>(IR/HR)	p=0.2709<br>(n=40)	p=0.8645<br>(n=45)	p=0.5674<br>(n=42)	p=0.2472<br>(n=42)
Tumor stage<br>(Stages I–III/Stage IV)	p=0.7537<br>(n=51)	p=0.4324<br>(n=58)	p=0.5373<br>(n=55)	p=0.4013<br>(n=51)
MYCN amplification<br>(+/−)	p=0.2772<br>(n=47)	p=0.4279<br>(n=56)	p=0.3328<br>(n=53)	p=0.8803<br>(n=51)
Shimada classification<br>(favorable/unfavorable)	p=0.8595<br>(n=55)	p=0.2776<br>(n=65)	p=0.9438<br>(n=62)	p=0.5789<br>(n=58)

Legend: P < 0.05 values are shown in bold and were considered statistically significant. F, female; M, male; HR, high risk; IR, intermediate risk; n, number of samples.

Table S4. Target genes of miR-141-3p, miR-150-5p, miR-181a-5p, and miR-182-5p selected using the TarBase v.9 and miRTarBase v.9 databases, by strong experimental evidence (luciferase assay, qPCR, or Western blot)

**miR-141-3p**	**miR-150-5p**	**miR-181a-5p**	**miR-182-5p**
BCL2	*PF4*	*ATM*	*AURKA*
CCND1	*MYB*	*BCL2*	*BARD1*
CCNE2	*TP53*	*CDKN1A*	*BCL2*
CDK2	*VEGFA*	*HRAS*	*BRCA1*
EDN1		*KRAS*	*CCND1*
PPARA		*MAPK1*	*CDK4*
PTEN		*MCL1*	*CDKN1A*
TIAM1		*NRAS*	*CHEK2*
		PTEN	*FBXW7*
		PTPN11	*PTEN*
		VEGFA	*RB1*
			TIAM1
			TP53

Table S5. Correlation between miRNAs and target genes expression (Pearson correlation).

miR-141-3p					
	n	r	r^2	equation	P
AURKA	14	0.2097	0.04399	Y = 0.1458*X – 9.161	0.4717
BCL2	15	0.01283	0.0001647	Y = 0.006145*X – 10.66	0.9638
FBXW7	17	-0.1830	0.03349	Y = -0.06652*X – 11.15	0.4820
MCL1	15	-0.002991	8.946e-006	Y = -0.001956*X – 10.72	0.9916
MYCN	16	-0.3230	0.1044	Y = -0.1126*X – 11.48	0.2223

miR-150-5p					
	n	r	r^2	equation	P
AURKA	14	0.1458	0.02125	Y = 0.1859*X – 0.2779	0.6190
BCL2	15	0.2491	0.06204	Y = 0.2282*X – 0.3435	0.3707
FBXW7	17	0.08923	0.007962	Y = 0.07366*X – 2.003	0.7334
MCL1	15	0.1379	0.01902	Y = 0.1725*X – 1.008	0.6240
MYCN	16	-0.4283	0.1834	Y = -0.3387*X – 4.733	0.0979

miR-181a-5p					
	n	r	r^2	equation	P
AURKA	14	0.01542	0.0002378	Y = 0.01193*X – 5.804	0.9583
BCL2	15	0.4362	0.1903	Y = 0.2350*X – 4.162	0.1040
FBXW7	17	-0.2855	0.08151	Y = -0.1292*X – 6.710	0.2667
MCL1	15	-0.1907	0.03637	Y = -0.1403*X – 6.896	0.4960
MYCN	**16**	**0.6175**	**0.3813**	**Y = 0.2667*X – 4.054**	**0.0108***

miR-182-5p					
	n	r	r^2	equation	P
AURKA	14	0.03597	0.001294	Y = 0.02524*X – 11.80	0.9028
BCL2	15	0.1014	0.01028	Y = 0.05983*X – 11.40	0.7192
FBXW7	17	-0.3555	0.1264	Y = -0.1997*X – 12.93	0.1614
MCL1	15	-0.2740	0.07509	Y = -0.2208*X – 13.29	0.3230
MYCN	16	-0.1466	0.0215	Y = -0.07866*X – 12.14	0.5879

Legend: n: number of samples

Table S6. Association between miRNA and target gene expression and prognostic parameters of the patients (frozen tumor samples).

**Parameter**	**miR-141-3p**	**miR-150-5p**	**miR-181a-5p**	**miR-182-5p**	**AURKA**	**BCL2**	**FBXW7**	**MCL1**	**MYCN**
Age (≥18 mo/<18 mo)	p=0.8836 (n=17)	p=0.5908 (n=17)	p=0.2161 (n=17)	p=0.8836 (n=17)	p=0.1119 (n=14)	p>0.9999 (n=15)	p=0.1331 (n=17)	p>0.9999 (n=15)	p=0.8868 (n=17)
Gender (F/M)	p=0.5362 (n=17)	p=0.4747 (n=17)	p=0.4747 (n=17)	p=0.2698 (n=17)	p>0.9999 (n=14)	p=0.2198 (n=15)	p>0.9999 (n=17)	p=0.6889 (n=15)	p=0.3502 (n=17)

Tumor stage (I, IV, IVS)	p=0.4818 (n=12)	p=0.7273 (n=12)	p=0.4818 (n=12)	p=0.6000 (n=12)	p=0.3810 (n=9)	p=0.1333 (n=11)	p=0.1455 (n=12)	p=0.3833 (n=10)	p=0.1455 (n=12)
MYCN amplification (+/−)	p=0.5185 (n=14)	p=0.6064 (n=14)	p>0.9999 (n=14)	p=0.6993 (n=14)	p=0.2020 (n=12)	p=0.4351 (n=13)	p=0.2977 (n=14)	p=0.6216 (n=13)	**p=0.0190** (n=14)
Risk group (HR, IR, LR)	p=0.1979 (n=16)	p=0.2952 (n=16)	p=0.3987 (n=16)	p=0.7151 (n=16)	p=0.4029 (n=13)	**p=0.0072** (n=15)	p=0.1059 (n=16)	p=0.2766 (n=14)	p=01417 (n=16)

Legend**:** Values are p-values followed by the number of samples included in each analysis. Statistically significant p-values are shown in bold. F, female; M, male; HR, high risk; IR, intermediate risk; LR, low risk; n, number of samples.

Supplementary figures

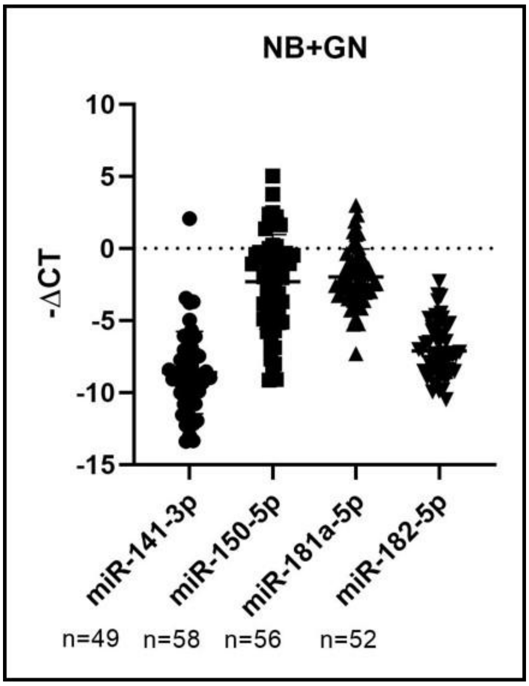

Figure S1: Expression of the four-miRNA panel relative to the endogenous control gene (RNU48) in the neuroblastoma (NB) and ganglioneuroma (GN) FFPE samples**.**

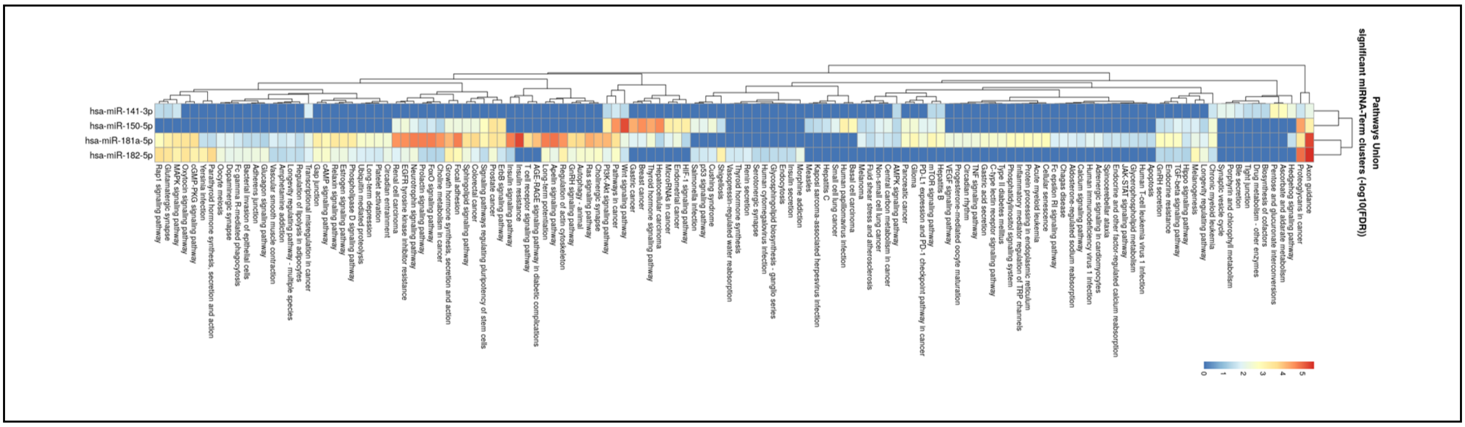

Figure S2. Heatmap, based on the pathways union function, showing the 132 signaling pathways regulated by the panel of the four miRNAs. The colors represent the involvement level of each miRNA based on the p value (Diana tools/ miRPath, v.4.0).

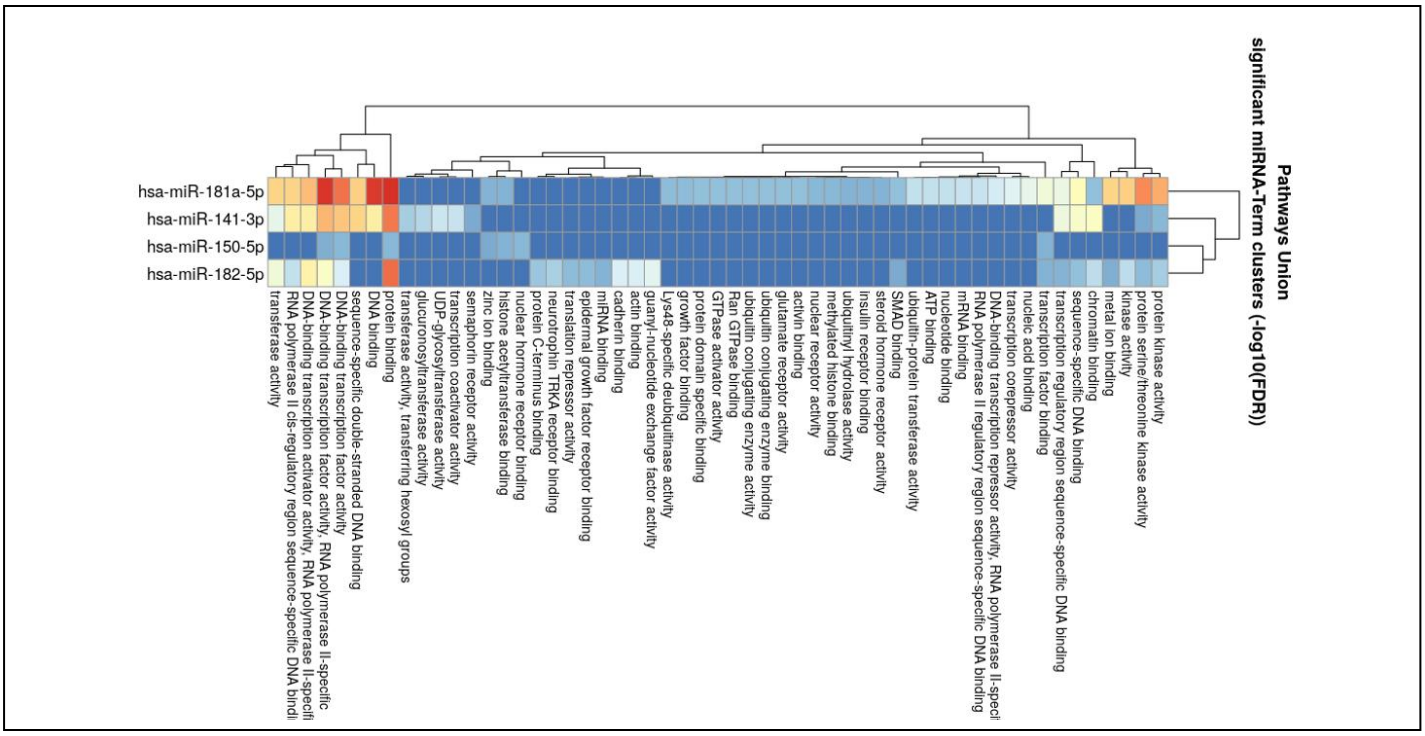

Figure S3. Heatmap, based on the *Gene Ontology* (GO) category (union function), showing the 55 biological process (p<0.05) regulated by the panel of the four miRNAs. The colors represent the involvement level of each miRNA based on the p value (Diana tools/ miRPath, v.4.0).

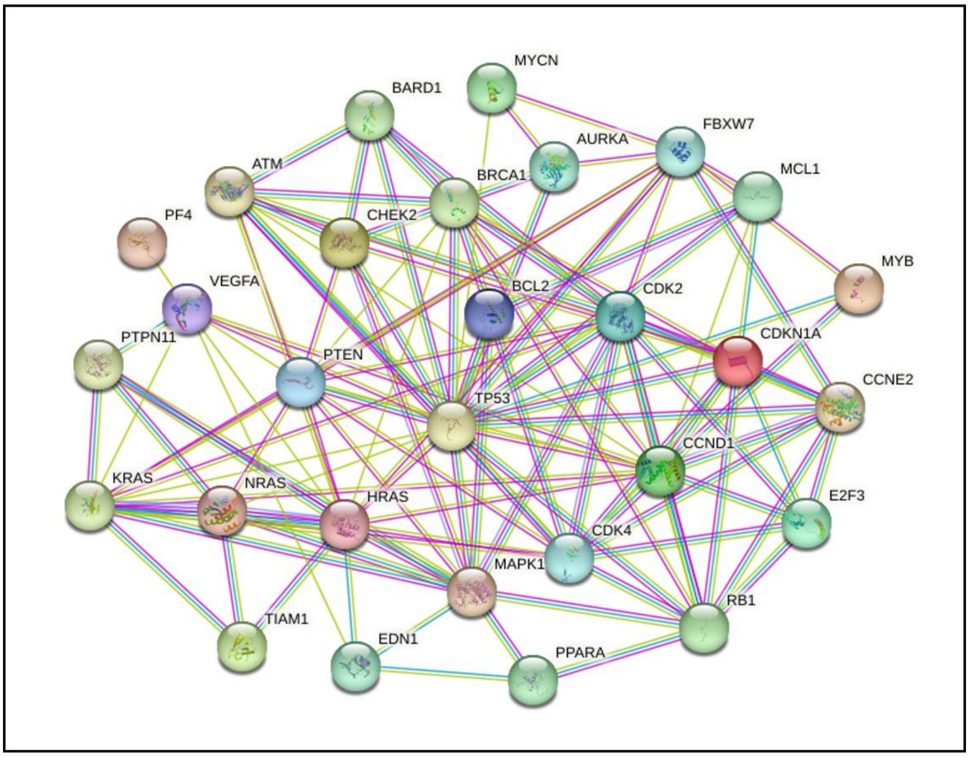

Figure S4. Protein-Protein Interaction (PPI) corresponds to the target genes of miR-141-3p, miR-150-5p, miR-181a-5p and miR-182-5p and the MYCN protein. Blue, pink and yellow lines indicate known interactions through databases, experimental methods and text mining, respectively (STRING v.12).

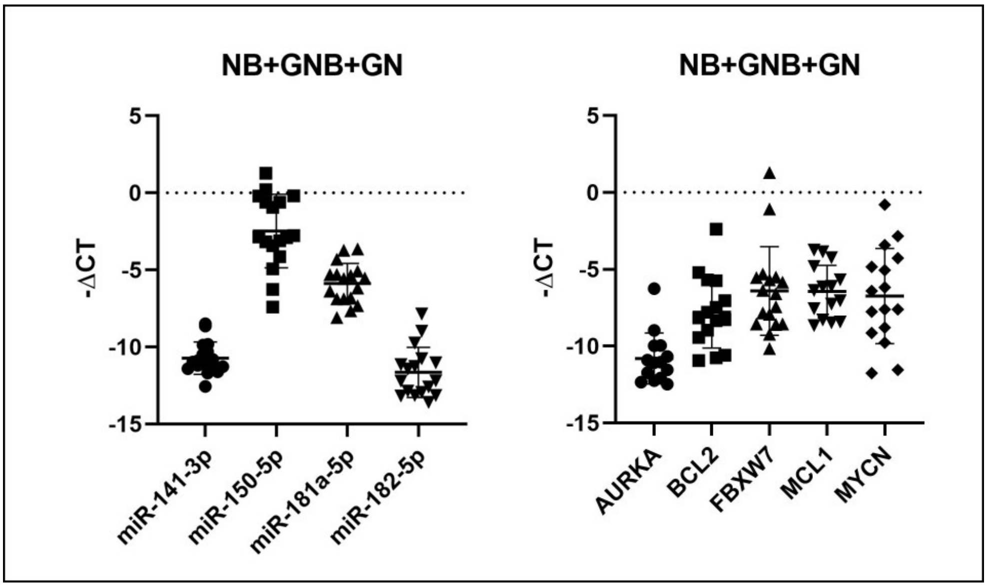

Figure S5. Expression of the four miRNA panel (A) and the selected genes (B) relative to the endogenous control genes (RNU48 (A) and *ACTB* and *GAPDH* mean (B), in the neuroblastoma (NB), ganglioneuroblastoma (GNB) and ganglioneuroma (GN) frozen samples**.**
